# Prevalence and Trends of Sexual Behaviors Among Young Adolescents Aged 12 Years to 15 Years in Low and Middle-Income Countries: Population-Based Study

**DOI:** 10.2196/45236

**Published:** 2023-06-07

**Authors:** Zhengyue Jing, Jie Li, Yi Wang, Chengchao Zhou

**Affiliations:** 1 Department of Social Security School of Health Policy and Management Nanjing Medical University Nanjing China; 2 Centre for Health Management and Policy Research School of Public Health, Cheeloo College of Medicine Shandong University Jinan China; 3 National Health Commission of China Key Lab of Health Economics and Policy Research Shandong University Jinan China; 4 Institute of Health and Elderly Care Shandong University Jinan China

**Keywords:** risky sexual behaviors, early sexual intercourse, multiple sexual partners, condom use, young adolescents, low and middle-income countries

## Abstract

**Background:**

Risky sexual behaviors remain significant public health challenges among adolescents. Nearly 90% of adolescents live in low and middle-income countries (LMICs), but few studies have used standardized methodology to monitor the prevalence and trends of sexual behaviors among adolescents in LMICs.

**Objective:**

This study aimed to assess the prevalence of sexual behaviors (ever had sexual intercourse, multiple sexual partners, and condom use) among adolescents aged 12 years to 15 years as well as the trends in prevalence between 2003 and 2017.

**Methods:**

For this population-based study, we used recent data from the Global School-based Student Health Survey conducted in 69 LMICs from 2003 to 2017 to assess the recent prevalence of sexual behaviors by using complex analysis and a random effects meta-analyses method. Using the chi-square trend test, we also assessed the trends in the prevalence of sexual behaviors in 17 countries that had conducted ≥1 round of surveys from 2003 to 2017.

**Results:**

We included 145,277 adolescents aged 12 years to 15 years (64,719/145,277, 44.5% boys) from the 69 LMICs that had conducted ≥1 survey and 80,646 adolescents aged 12 years to 15 years (34,725/80,646, 43.1% boys) from the 17 LMICs that had conducted ≥1 round of surveys. The recent global prevalence of ever had sexual intercourse was 6.9% (95% CI 6.2%-7.6%) and was higher among boys (10.0%, 95% CI 9.1%-11.1%) than girls (4.2%, 95% CI 3.7%-4.7%) and among those aged 14 years to 15 years (8.5%, 95% CI 7.7%-9.3%) than those aged 12 years to 13 years (4%, 95% CI 3.4%-4.7%). Among adolescents who had ever had sex, the recent global prevalence of having multiple sexual partners was 52% (95% CI 50.4%-53.6%) and was higher among boys (58%, 95% CI 56.1%-59.9%) than girls (41.4%, 95% CI 38.9%-43.9%) and among those aged 14 years to 15 years (53.5%, 95% CI 51.6%-55.4%) than those aged 12 years to 13 years (49.7%, 95% CI 45.9%-53.5%). Among adolescents who had ever had sex, the recent global prevalence of condom use was 58.1% (95% CI 56.2%-59.9%) and was higher among girls (59.2%, 95% CI 56.4%-61.9%) than boys (57.7%, 95% CI 55.7%-59.7%) and among those aged 14 years to 15 years (59.9%, 95% CI 58.0%-61.8%) than those aged 12 years to 13 years (51.6%, 95% CI 47.5%-55.7%). Between the earliest and latest surveys, the overall prevalence of ever had sexual intercourse (3.1% decrease) and condom use (2.0% decrease) showed downward trends. The overall prevalence of having multiple sexual partners increased by 2.6%.

**Conclusions:**

We provide evidence and important implication for policymakers to develop targeted policy support systems to prevent and reduce risky sexual behaviors among young adolescents in LMICs with a high prevalence of risky sexual behaviors.

## Introduction

Adolescence is a period of rapid change in physical, psychological, emotional, and cognitive development, during which adolescents become more interested in sexual behaviors. Although sexual behaviors are widely considered a normative and physiological component of adolescent development, risky sexual behaviors including early initiation of sexual intercourse, having multiple sexual partners, and condom nonuse remain significant public health challenges among adolescents due to their potentially deleterious effects on later sexual and reproductive health [1]. Sexual debut is not deterministic of later sexual activities and risks, but sexual intercourse initiated at an earlier than normative age (typically defined as 15 years or younger) exposes adolescents, particularly adolescent girls, to a variety of risks such as HIV infection and other sexually transmitted infections (STIs) [2,3]. Girls who initiate sexual intercourse early were found to be at higher risk of reporting unintended pregnancies and STIs than those who delay intercourse until late adolescence [4,5]. Early sexual behavior is a public health concern among adolescents both in low and middle-income countries (LMICs) and high-income countries (HICs), but the adverse consequences of early sexual debut for adolescents in LMICs are more severe than for those in HICs. For example, previous studies have found that adolescent birth rates in LMICs are more than double that of HICs and most STIs occur in LMICs [6,7]. In addition, early sexual intercourse has been associated with reporting negative social and psychological outcomes (such as suicidal behaviors) [8] and subsequent higher-risk behaviors including alcohol or drug use during sex, lower levels of condom use, and forced sex [9,10].

Having multiple sexual partners is a common practice among young people. A review of data from the Global School-based Student Health Survey (GSHS) in 21 countries between 2010 and 2016 found that 53.1% of adolescents aged 12 years to 15 years who had sexual intercourse reported having multiple sexual partners [11]. Having multiple sexual partners is a risky sexual behavior that increases the risk of HIV and other STI transmission among adolescents [12]. A previous study indicated that the high prevalence of HIV/AIDS in sub-Saharan Africa is driven by high levels of multiple sexual partnerships [13].

Condom use has been acknowledged as an effective way to reduce the risk of gonorrhea, herpes simplex virus type 2, syphilis, and other STIs [14]. A review indicated that consistent use of condoms could effectively reduce the incidence of HIV/AIDS by 80% [15]. Meanwhile, condom use was associated with a lower risk of unintended pregnancy among adolescents, and the failure rate of male condoms for unintended pregnancy was about 2% [16]. Despite this, the prevalence of condom use among adolescents remains low. A study reported that nearly two-thirds of adolescents aged 15 years to 21 years did not use condoms during their last sexual intercourse [17].

Healthy sexuality is a key component of adolescent development. Nearly 90% of adolescents live in LMICs, and monitoring the prevalence and trends of sexual behaviors among adolescents in LMICs can help public health and education sectors design appropriate prevention and intervention strategies to promote sexual and reproductive health in adolescents. Although previous studies have reported the prevalence of sexual behaviors among adolescents aged 13 years to 15 years in some countries [18], as more countries have been released in the GSHS data set, there is an urgent need to analyze more up-to-date data and examine the difference in the prevalence of sexual behaviors among young adolescents. Differences in the prevalence of adolescent sexual behavior across countries may be influenced by race, religion, the society’s culture, and economic status. Age and gender are also important factors to consider. However, few studies have used standardized questionnaires to examine and compare the age and gender differences in the prevalences of risky sexual behaviors among young adolescents in LMICs. More importantly, the most effective policies to prevent risky sexual behaviors may change over time; therefore, identifying trends in the prevalence of sexual behaviors is important for these countries to understand the effectiveness of previously developed policies in combating risky sexual behaviors among young adolescents and thus adjust intervention policies to fit the changes in policy needs and impacts. Therefore, it is imperative to analyze trends in the prevalences of risky sexual behaviors among young adolescents to provide evidence for the development of intervention programs. However, to our knowledge, few previous studies have assessed recent trends in the prevalences of risky sexual behaviors among young adolescents in LMICs.

Therefore, this study aimed to use recent data collected in the GSHS from 2003 to 2017 to assess the prevalences of sexual behaviors including ever had sexual intercourse, multiple sexual partners, and condom use as well as their age and gender differences among adolescents aged 12 years to 15 years in 69 LMICs. We also aimed to evaluate trends in the prevalences of sexual behaviors among adolescents in 17 LMICs between 2003 and 2017.

## Methods

### Study Design and Participants

This study used the latest GSHS data (2003-2017) publicly available on the websites of the US Centers for Disease Control and Prevention (CDC) and World Health Organization (WHO). Developed by the WHO and US CDC, the GSHS is a school-based self-administered survey for young adolescents aged 12 years to 15 years. The goal of the GSHS is to assess the health behavior risks and protective factors of middle-school students across countries and ultimately help countries develop and provide health care programs, resources, and policies that promote the health of adolescents [19]. To ensure the comparability of data across countries, the GSHS survey used the same standardized procedure (including sampling strategy, study methodology, and questionnaire) in each country; the GSHS questionnaire was translated into the local language, and each country was free to select question modules.

The selection of respondents in each country participating in the GSHS was based on a 2-stage random cluster sampling procedure. In the first stage, schools were randomly selected from all middle schools in each country by using the probability proportionate to size sampling method. In the second stage, classes were randomly selected from each selected school. All the students in each selected class were eligible to participate in this survey. Finally, based on the available data on sexual behaviors in the GSHS database, for the analysis of the prevalence of sexual behaviors, this study used the most recent GSHS data from 69 countries in 6 WHO regions (including 17 from Africa, 30 from the Americas, 1 from the Eastern Mediterranean, 2 from Europe, 5 from Southeast Asia, and 14 from the Western Pacific) that had conducted at least one survey between 2003 and 2017. To analyze the trends in the prevalences of sexual behaviors, we used the GSHS data from 17 countries in 4 WHO regions (5 from Africa, 7 from the Americas, 2 from Southeast Asia, and 3 from the Western Pacific) that had conducted more than one round of surveys between the earliest and latest surveys.

### Outcomes and Definitions

Sexual intercourse was measured in this study using the question: “Have you ever had sexual intercourse?” The answer was dichotomized as “Yes” or “No.” Sexual partners were measured in this study using the question: “During your life, with how many people have you had sexual intercourse?” Multiple sexual partners were defined as having 2 or more sexual partners during their lifetime. Condom use was measured in this study using the question: “The last time you had sexual intercourse, did you or your partner use a condom?” The answer was also dichotomized as “Yes” or “No.” The prevalences of multiple sexual partners and condom use were calculated for those having sexual intercourse.

### Statistical Analysis

The complex sampling command in SPSS Version 22.0 (IBM Corp) was used to conduct the statistical analysis. To adjust the sampling survey method and the differences between sampled students and national students, all complex sample analyses in this study were performed using 3 weighted variables included in each GSHS data set: strata, primary sampling unit, and weights. Weighted prevalences and corresponding 95% CIs of ever had sexual intercourse, having had multiple sexual partners, and condom use were calculated by region and country and by sex and age. ArcGIS software was used to map the prevalence of sexual behaviors among adolescents in each country. Chi-square analysis was used to test for differences in the prevalences between sexes and age groups, and *P* values <.05 indicated that the difference was statistically significant. The chi-square trend test was used to test the secular trend in the prevalences between the earliest survey and latest survey, and *P* values <.05 represented significant downward or upward trends in prevalences over time. Meanwhile, due to the significant heterogeneity between countries, we used the random effects model to calculate the pooled overall and regional prevalences of ever had sexual intercourse through the meta-analysis module in Stata Version 11.0.

### Ethical Considerations

The ethics committee (usually the Ministry of Health or Education) of each country included in the GSHS survey reviewed and approved the study protocol. This survey was anonymous, and the answers were protected by privacy law. All participants and their parents gave their informed written or verbal consent for participation prior to the survey.

## Results

### Participants

As shown in Table 1 145,277 adolescents aged 12 years to 15 years were included from 69 countries in 6 WHO regions that had conducted at least one survey between 2003 and 2017. The sample sizes ranged from 218 in Nauru to 18,031 in Argentina. A total of 80,646 adolescents aged 12 years to 15 years were included from 17 countries in 4 WHO regions that had conducted more than one round of surveys between 2003 and 2017.

**Table 1:** Survey characteristics of the Global School-based Student Health Surveys of adolescents aged 12 years to 15 years by country, 2003-2017.

Country		Survey year	Total survey sample, N	Response rate, n (%)
**Africa**				
	Benin	2016	712	625 (87.8)
	Botswana	2005	1391	1016 (73.0)
	**Eswatini**			
		2003	6664	4558 (68.4)
		2013	1314	1089 (82.9)
	**Ghana**			
		2007	4235	2520 (59.5)
		2012	1330	861 (64.7)
	Liberia	2017	529	298 (56.3)
	Malawi	2009	2185	1649 (75.5)
	Mauritania	2010	1272	865 (68.0)
	**Mauritius**			
		2011	2071	2032 (98.1)
		2017	1946	1515 (77.9)
	Mozambique	2015	652	445 (68.3)
	**Namibia**			
		2004	4492	2226 (49.6)
		2013	1918	1385 (72.2)
	Senegal	2005	2633	2096 (79.6)
	**Seychelles**			
		2007	1146	861 (75.1)
		2015	2060	1535 (74.5)
	Sierra Leone	2017	1835	1101 (60)
	Tanzania	2014	2590	1979 (76.4)
	Uganda	2003	1868	1238 (66.3)
	Zambia	2004	1315	382 (29)
	Zimbabwe	2003	3877	2569 (66.3)
**Americas**				
	**Anguilla**			
		2009	696	546 (78.4)
		2016	571	448 (78.4)
	Antigua and Barbuda	2009	1198	1028 (85.8)
	**Argentina**			
		2007	1523	1292 (84.8)
		2012	21,620	18,031 (83.4)
	Bahamas	2013	1304	1034 (79.3)
	Barbados	2011	1502	1240 (82.6)
	Belize	2011	1597	1345 (84.2)
	Bolivia	2012	2761	2318 (83.9)
	British Virgin Islands	2009	1191	1008 (84.6)
	Cayman	2007	1265	915 (72.3)
	Chile	2013	1342	1182 (88.1)
	Colombia	2007	7963	7187 (90.3)
	Costa Rica	2009	2259	2023 (89.6)
	Curaçao	2015	1491	1261 (84.6)
	Dominica	2009	1308	1047 (80)
	Ecuador	2007	4508	3711 (82.3)
	El Salvador	2013	1600	1438 (89.9)
	Grenada	2008	1296	1018 (78.5)
	**Guatemala**			
		2009	4461	4002 (89.7)
		2015	3591	2880 (80.2)
	**Guyana**			
		2004	1060	855 (80.7)
		2010	1958	1519 (77.6)
	Honduras	2012	1474	1253 (85)
	Jamaica	2017	1057	885 (83.7)
	Paraguay	2017	1972	1720 (87.2)
	Peru	2010	2350	2260 (96.2)
	Saint Kitts and Nevis	2011	1463	1206 (82.4)
	Saint Lucia	2007	1070	926 (86.5)
	Saint Vincent and the Grenadines	2007	1184	956 (80.7)
	**Suriname**			
		2009	1043	934 (89.5)
		2016	1448	1300 (89.8)
	**Trinidad and Tobago**			
		2011	2352	1961 (83.4)
		2017	2749	2205 (80.2)
	**Uruguay**			
		2006	2876	2542 (88.4)
		2012	2855	2587 (90.6)
	Venezuela	2003	3901	3052 (78.2)
**Eastern Mediterranean**				
	Djibouti	2007	961	655 (68.2)
**Europe**				
	Macedonia	2007	1538	1364 (88.7)
	Tajikistan	2006	7457	5789 (77.6)
**Southeast Asia**				
	Bhutan	2016	3268	2873 (87.9)
	**Indonesia**			
		2007	3013	2784 (92.4)
		2015	11,063	6992 (63.2)
	Nepal	2015	4565	3392 (74.3)
	**Thailand**			
		2008	2671	2309 (86.4)
		2015	4120	3399 (82.5)
	Timor-Leste	2015	2053	961 (46.8)
**Western Pacific**				
	Brunei Darussalam	2014	1822	1611 (88.4)
	Cambodia	2013	1809	1471 (81.3)
	**Fiji**			
		2010	1491	1229 (82.4)
		2016	1512	1184 (78.3)
	French Polynesia	2015	1898	1678 (88.4)
	Kiribati	2011	1337	1155 (86.4)
	Lao People's Democratic Republic	2015	1636	1453 (88.8)
	Malaysia	2011	16,248	13,880 (85.4)
	Mongolia	2013	3695	3303 (89.4)
	Nauru	2011	361	218 (60.4)
	**Samoa**			
		2011	2153	676 (31.4)
		2017	1054	707 (67.1)
	**Vanuatu**			
		2011	844	782 (92.7)
		2016	1278	900 (70.4)
	Tuvalu	2013	675	455 (67.4)
	Viet Nam	2013	1743	1605 (92.1)
	Wallis and Futuna	2015	713	601 (84.3)
**All countries combined**				
	More than 1 survey	—^a^	107,528	80,646 (75)
	Most recent survey	—	182,051	145,277 (79.8)

^a^Not applicable.

### Prevalences of Sexual Behaviors in Young Adolescents

**Figure 1:**
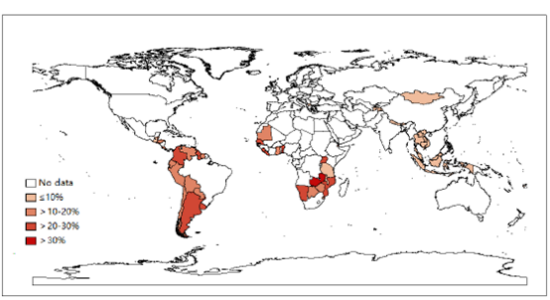
Prevalence of ever had sexual intercourse among young adolescents aged 12 years to 15 years based on the most recent Global School-based Student Health Survey from 69 countries, 2003-2017.

The overall prevalence of ever had sexual intercourse was 6.9% (95% CI 6.2%-7.6%) and was lowest in the Western Pacific and highest in the Americas (Table 2). Both gender and age differences were statistically significant in Africa, the Americas, and Southeast Asia. In Table S1 in Multimedia Appendix 1, nearly two-thirds (47 countries, 68%) of the 69 countries had a prevalence of ever had sexual intercourse >10%, while those in Europe and South Asia had a lower prevalence of <10% (Figure 1). Almost all countries had a higher prevalence for boys than girls, as well as for those aged 14 years to 15 years than those aged 12 years to 13 years.

**Table 2:** The prevalence of ever had sexual intercourse among young adolescents aged 12 years to 15 years by World Health Organization region, sex, and age in 2003-2017.

Region	Total sample, % (95% CI)	Boys, % (95% CI)	Girls, % (95% CI)	*P* value	12-13 years old, % (95% CI)	14-15 years old, % (95% CI)	*P* value
Total	6.9 (6.2-7.6)	10.0 (9.1-11.1)	4.2 (3.7-4.7)	<.001	4.0 (3.4-4.7)	8.5 (7.7-9.3)	<.001
Africa	12.9 (11.4-14.6)	20.1 (17.7-22.9)	6.2 (5.2-7.3)	<.001	8.7 (7.0-10.6)	15.2 (13.5-17.2)	<.001
Americas	17.8 (16.9-18.9)	24.4 (23.0-25.8)	12.1 (11.1-13.1)	<.001	9.5 (8.6-10.4)	22.2 (21.0-23.5)	<.001
Eastern Mediterranean	15.0 (12.0-18.6)	22.2 (17.9-27.1)	4.8 (3.1-7.5)	<.001	10.3 (4.5-21.9)	16.0 (13.3-19.0)	.24
Europe	3.0 (2.3-4.0)	5.1 (3.8-6.8)	0.9 (0.5-1.5)	<.001	2.4 (1.4-4.1)	3.2 (2.4-4.4)	.30
Southeast Asia	1.8 (1.4-2.3)	2.6 (2.0-3.4)	1.1 (0.8-1.6)	<.001	1.1 (0.7-1.6)	2.5 (2.0-3.1)	<.001
Western Pacific	1.2 (0.9-1.6)	1.5 (1.1-2.1)	0.9 (0.6-1.3)	.02	0.9 (0.6-1.2)	1.2 (0.9-1.7)	.11

**Figure 2:**
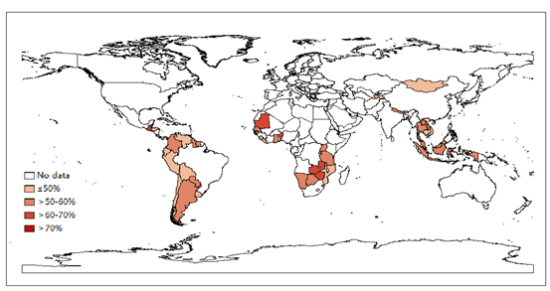
Prevalence of having multiple sexual partners among young adolescents aged 12 years to 15 years who ever had sexual intercourse based on the most recent Global School-based Student Health Survey from 69 countries, 2003-2017.

The prevalences of having multiple sexual partners and condom use among adolescents were based on those who ever had sexual intercourse. A total of 20,257 adolescents were included in the analysis of the prevalences of having multiple partners and condom use. The overall prevalence of having multiple sexual partners was 52.0% (95% CI 50.4%-53.6%) and was lowest in Western Pacific and highest in the Eastern Mediterranean (Table 3). The gender difference was statistically significant in all regions except for the Eastern Mediterranean, and the age difference was significant only in the Americas. In Table S2 in Multimedia Appendix 1, most (50 countries, 72%) of the 69 included countries had a prevalence of having multiple sexual partners >50% (Figure 2). The prevalence ranged from 2 times to 9 times higher among boys than among girls across countries, and the prevalence in adolescents aged 14 years to 15 years was higher than in those aged 12 years to 13 years in 47 of the 61 countries (data were unavailable for 8 countries in the Western Pacific).

**Table 3:** The prevalence of having multiple sexual partners among young adolescents aged 12 years to 15 years by World Health Organization region, sex, and age in 2003-2017.

Region	Total sample, % (95% CI)	Boys, % (95% CI)	Girls, % (95% CI)	*P* value	12-13 years old, % (95% CI)	14-15 years old, % (95% CI)	*P* value
Total	52.0 (50.4-53.6)	58.0 (56.1-59.9)	41.4 (38.9-43.9)	<.001	49.7 (45.9-53.5)	53.5 (51.6-55.4)	.12
Africa	52.3 (49.3-55.3)	54.7 (51.2-58.2)	45.2 (39.5-51.0)	.007	54.4 (46.7-61.9)	51.7 (47.9-55.5)	.59
Americas	52.3 (50.6-54.0)	59.9 (57.9-61.9)	38.7 (35.9-41.6)	<.001	46.9 (42.8-51.0)	53.5 (51.5-55.5)	.008
Eastern Mediterranean	71.2 (61.1-79.5)	72.3 (62.2-80.5)	63.9 (37.4-83.9)	.48	63.2 (39.1-82.2)	72.2 (62.1-80.4)	.40
Europe	40.8 (34.7-47.3)	44.8 (37.6-52.1)	16.4 (7.9-30.9)	.001	41.1 (28.1-55.4)	40.8 (34.0-47.9)	.97
Southeast Asia	56.0 (49.1-62.7)	60.4 (51.8-68.5)	47.5 (37.4-57.7)	.04	47.3 (34.0-61.0)	59.4 (51.6-66.6)	.13
Western Pacific	32.3 (22.1-44.4)	57.4 (51.9-62.9)	40.5 (34.0-47.4)	.001	53.0 (42.8-63.0)	51.7 (47.1-56.2)	.82

**Figure 3:**
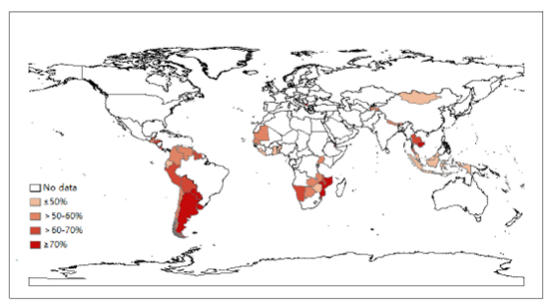
Prevalence of condom use at last sex among young adolescents aged 12 years to 15 years who ever had sexual intercourse based on the most recent Global School-based Student Health Survey from 69 countries, 2003-2017.

The overall prevalence of condom use at last sex was 58.1% (95% CI 56.2%-59.9%) and was lowest in the Western Pacific and highest in Europe (Table 4). The overall prevalence of condom use at last sex exceeded 50% in all regions, regardless of gender and age, except for among boys in Africa and the Western Pacific. In Table S3 in Multimedia Appendix 1, more than three-quarters (52 countries, 75%) of the included 69 countries had a prevalence >50% (Figure 3). In 38 of the 62 countries (data were unavailable for 7 countries in the Western Pacific), the prevalence in boys was higher than in girls, particularly in all countries in the Western Pacific. The prevalence in adolescents aged 14 years to 15 years was higher than in those aged 12 years to 13 years in 40 of the 62 countries, while all countries in Europe showed the opposite result.

**Table 4:** The prevalence of condom use at last sex among young adolescents aged 12 years to 15 years by World Health Organization region, sex, and age in 2003-2017.

Region	Total sample, % (95% CI)	Boys, % (95% CI)	Girls, % (95% CI)	*P* value	12-13 years old, % (95% CI)	14-15 years old, % (95% CI)	*P* value
Total	58.1 (56.2-59.9)	57.7 (55.7-59.7)	59.2 (56.4-61.9)	.35	51.6 (47.5-55.7)	59.9 (58.0-61.8)	<.001
Africa	47.8 (43.4-52.2)	45.9 (41.5-50.4)	53.4 (46.3-60.3)	.03	37.9 (30.7-45.7)	50.9 (46.4-55.3)	.002
Americas	65.2 (63.2-67.2)	66.1 (63.7-68.3)	63.8 (60.6-66.8)	.19	60.8 (56.7-64.7)	66.2 (63.9-68.5)	.02
Eastern Mediterranean	64.1 (51.6-75.0)	64.9 (51.4-76.3)	59.4 (31.5-82.3)	.72	64.6 (40.9-82.7)	64.1 (51.0-75.4)	.97
Europe	71.7 (65.2-77.4)	71.3 (64.5-77.3)	73.9 (62.2-83.0)	.62	72.9 (52.7-86.7)	71.4 (64.1-77.7)	.87
Southeast Asia	54.8 (48.6-60.9)	56.7 (48.5-64.5)	51.3 (41.5-61.0)	.36	55.7 (43.2-67.5)	54.5 (46.8-62.0)	.86
Western Pacific	47.6 (33.2-62.3)	49.0 (44.3-53.8)	31.0 (22.6-40.8)	<.001	44.4 (34.2-55.2)	42.8 (37.5-48.3)	.77

### Trends in the Prevalences of Sexual Behaviors in Young Adolescents

As shown in Table 5, the overall prevalence of ever had sexual intercourse in young adolescents showed a downward trend over time between the earliest and latest surveys (3.1% decrease); a similar trend was observed by sex (boys, 3.9% decrease; girls, 2.5% decrease) and age group (12-13 years, 1.1% decrease; 14-15 years, 3.7% decrease). Specifically, in Table S4 in Multimedia Appendix 1, the prevalence of ever had sexual intercourse decreased in 9 countries (with the largest decrease of 24.6% in Samoa) and increased in 8 countries (with the largest increase of 7.9% in Seychelles).

**Table 5:** The trends in the prevalence of ever had sexual intercourse among young adolescents aged 12 years and 15 years between 2003 and 2017 or the earliest and latest surveys by World Health Organization region, sex, and age.

Region	Total sample, % (95% CI)	*P* value	Boys, % (95% CI)	*P* value	Girls, % (95% CI)	*P* value	12-13 years old, % (95% CI)	*P* value	14-15 years old, % (95% CI)	*P* value
Total	–3.1 (–3.5 to –2.7)	<.001	–3.9 (–4.5 to –3.3)	<.001	–2.5 (–2.9 to –2.1)	<.001	–1.1 (–2.0 to –0.2)	.02	–3.7 (–4.2 to –3.2)	<.001
Africa	–3.4 (–4.5 to –2.3)	.008	–2.8 (–4.7 to –0.9)	<.001	–3.7 (–4.9 to –2.5)	<.001	–3.9 (–7.8 to 0.1)	.31	–3.1 (–4.3 to –1.9)	.001
Americas	–2.6 (–3.5 to –1.7)	<.001	–6.6 (–8.1 to –5.1)	<.001	0 (–1.1 to 1.1)	.99	1.3 (–0.8 to 3.4)	.43	–2.6 (–3.8 to –1.4)	<.001
Southeast Asia	–1.0 (–1.5 to –0.5)	.008	–0.8 (–1.6 to 0.1)	.99	–1.2 (–1.8 to –0.6)	.002	–0.5 (–3.5 to 2.5)	.92	–1.2 (–1.9 to –0.5)	.02
Western Pacific	–3.2 (–4.7 to –1.7)	<.001	–4.6 (–7.7 to –1.5)	<.001	–1.9 (–3.2 to –0.6)	<.001	–3.1 (–10.6 to 4.4)	.32	–5.3 (–7.2 to –3.4)	<.001

A total of 13,051 adolescents were included in the trend analyses of the prevalences of having multiple partners and condom use. Table 6 shows that the overall prevalence of having multiple sexual partners increased by 2.6% over time; the upward trend was similar among girls and adolescents aged 14 years to 15 years (girls, 5.8% increase; age 14-15 years, 2.7% increase). All regions experienced an increase in the prevalence of having multiple sexual partners except for Africa. Specifically, in Table S5 in Multimedia Appendix 1, the prevalence of having multiple sexual partners increased in 9 countries (with the largest increase of 14.2% in Namibia), was unchanged in 2 countries, and decreased in 5 countries (with the largest decrease of 3.9% in Guyana).

**Table 6:** The trends in the prevalence of having multiple sexual partners among young adolescents aged 12 years to 15 years between 2003 and 2017 or the earliest and latest surveys by World Health Organization region, sex, and age.

Region	Total sample, % (95% CI)	*P* value	Boys, % (95% CI)	*P* value	Girls, % (95% CI)	*P* value	12-13 years old, % (95% CI)	*P* value	14-15 years old, % (95% CI)	*P* value
Total	2.6 (0.8 to 4.4)	<.001	0.4 (–1.8 to 2.6)	.56	5.8 (2.9 to 8.7)	.047	2.5 (–1.9 to 6.9)	.81	2.7 (0.7 to 4.7)	<.001
Africa	–0.2 (–3.9 to 3.5)	.95	–0.6 (–5.3 to 4.1)	.89	–0.5 (–6.2 to 5.2)	.93	27.5 (20.7 to 34.3)	<.001	–9.6 (–13.8 to –5.4)	.002
Americas	1.6 (–0.9 to 4.1)	.59	–0.5 (–3.5 to 2.5)	.06	5.9 (1.8 to 10.0)	<.001	1.0 (–5.1 to 7.1)	.26	2.0 (–0.7 to 4.7)	.07
Southeast Asia	7.4 (–3.9 to 18.7)	.25	4.4 (–10.1 to 18.9)	.42	8.2 (–8.1 to 24.5)	.38	–22.9 (–45.8 to 0.1)	.63	14.9 (2.2 to 27.6)	.046
Western Pacific	4.7 (–3.8 to 13.2)	.58	3.7 (–6.6 to 14.0)	.86	6.3 (–8.5 to 21.1)	.09	–8.9 (–37.0 to 19.2)	.15	4.9 (–4.1 to 13.9)	.80

In Table 7, the overall prevalence of condom use at last sex decreased by 2.0% over time; the downward trend was similar in boys (4.5% decrease) but increased for girls (1.8% increase). The decrease was not significant in adolescents aged 12 years to 13 years or 14 years to 15 years. Specifically, in Table S6 in Multimedia Appendix 1, the prevalence of condom use at last sex decreased in 10 countries (with the largest decrease of 9.7% in Ghana) and increased in 6 countries (with the largest increase of 14.6% in Namibia).

**Table 7:** The trends in the prevalence of condom use among young adolescents aged 12 years to 15 years between 2003 and 2017 or the earliest and latest surveys by World Health Organization region, sex, and age.

Region	Total sample, % (95% CI)	*P* value	Boys, % (95% CI)	*P* value	Girls, % (95% CI)	*P* value	12-13 years old, % (95% CI)	*P* value	14-15 years old, % (95% CI)	*P* value
Total	–2.0 (–3.7 to –0.3)	<.001	–4.5 (–6.6 to –2.4)	<.001	1.8 (–1.0 to 4.6)	.67	–2.4 (–6.5 to 1.7)	.64	–1.8 (–3.6 to 0.1)	.55
Africa	–3.6 (–7.2 to 0.1)	.57	–7.3 (–12.1 to –2.5)	.004	3.5 (–2.2 to 9.2)	.74	–12.6 (–20.4 to –4.8)	<.001	–0.3 (–4.5 to 3.9)	.97
Americas	–4.2 (–6.3 to –2.1)	<.001	–5.6 (–8.1 to –3.1)	.001	–1.8 (–5.4 to 1.8)	.75	–11.9 (–17.0 to –6.8)	<.001	–2.9 (–5.2 to –0.6)	.43
Southeast Asia	6.8 (–4.1 to 17.7)	.57	5.7 (–8.2 to 19.6)	.88	6.6 (–10.3 to 23.5)	.50	18.8 (–3.1 to 40.7)	.38	4.0 (–8.5 to 16.5)	.85
Western Pacific	2.2 (–6.4 to 10.8)	.72	4.0 (–6.5 to 14.5)	.91	–4.0 (–18.9 to 10.9)	.44	22.3 (–4.5 to 49.1)	.21	1.1 (–8.0 to 10.2)	.37

## Discussion

### Principal Findings

This study used the most recent GSHS data from 69 countries that had conducted at least one survey between 2003 and 2017 to assess the prevalences of sexual behaviors among adolescents aged 12 years to 15 years. Specifically, the overall prevalence of ever had sexual intercourse was 6.9%, and among adolescents who had ever had sex, the overall prevalences of having multiple sexual partners and using condom at last sex were 52.0% and 58.1%, respectively.

The prevalences of sexual behaviors among young adolescents varied widely across countries, and these differences may be due to several factors, including the social cultural environment, economic status, policy, race, and attitudes toward sex. For example, we found that the lowest prevalences of ever had sexual intercourse were in Indonesia, Malaysia, and Tajikistan, which can be explained by the religious culture embedded in these countries. Most people in these countries embrace Islam, which prohibits premarital sex and regards sexuality as a taboo and sensitive subject [20]. Especially in Indonesia, as the largest Islamic country with legal restrictions on pornography in the world [21], adolescents were generally considered to be more sexually conservative and bound due to the strict legal regulations and Islamic values.

The prevalences of sexual behaviors in boys were higher than in girls in most LMICs, which was consistent with previous studies in various countries, including HICs [22]. This finding may be due to the difference in sexual development between boys and girls. Previous studies have suggested that boys are more likely to report risky sexual behaviors and to initiate sexual intercourse earlier than girls [23,24]. Meanwhile, boys have more permissive attitudes about premarital sexual activity, while sexual intercourse in girls is often accompanied by trust, love, and a romantic relationship [25]. On the other hand, there are double standards in society for early sexual intercourse behaviors between boys and girls. Boys experience more sexual freedom, and sexual initiation in boys is regarded as a symbol of masculinity or rite of passage in some countries [26]. However, premarital sexual intercourse is stigmatized or labeled as indiscreet in girls, especially in Asian countries [23], and girls not only are bearing multiple social pressures but also have a higher risk of reporting unintended pregnancy and poor reproductive health. It is worth noting that the prevalence of condom use at last sex among boys in LMICs (57.7%) is lower than that from data obtained from HICs such as the United States (61% in those aged 13-19 years) and Australia (65.1% in those aged 10-12 years) and that future measures are needed to increase the use of condoms among not only girls but also boys in LMICs [27,28].

This study also provided evidence that the prevalences of sexual behaviors among adolescents aged 14 years to 15 years were higher than among those aged 12 years to 13 years in most countries, which has been shown in many previous studies [29]. This can be interpreted using the normal sexual development process. The expression of secondary sex characteristics is influenced by sexual-related hormones in puberty. A study showed that, during the ages of 12 years to 15 years, changes in hormones mature the reproductive system with age [30]. We speculated that sexual development may arouse sexual desire; thus, older adolescents are more likely to engage in sexual intercourse than younger adolescents.

Based on the GSHS data from 17 countries that had conducted more than one round of survey between the earliest and latest surveys, the overall prevalences of ever had sexual intercourse and condom use at last sex decreased over time, but the prevalence of having multiple sexual partners increased over time. To our knowledge, few studies have investigated the trends in sexual behaviors among young adolescents in LMICs, and this is the first study to explore the trends of sexual behaviors in young adolescents aged 12 years to 15 years in LMICs. An earlier study based on both the Youth Risk Behavior Survey (YRBS) and National Survey of Adolescent Males data found that the proportion of male adolescents aged 15 years to 17 years reporting sexual intercourse decreased by 9% from 1991 to 1997 and by 8% from 1988 to 1995 [31]. Another YRBS report based on recent surveillance data from the United States found that the percentages of high school students who ever had sex, had multiple sexual partners, or used a condom at last sex declined from 2009 to 2019 [32], with the downward trend in sexual intercourse and condom use at last sex consistent with our findings.

From a public health perspective, the decline in the prevalence of sexual intercourse among young adolescents aged 12 years to 15 years was a salutary trend, as sexual intercourse at an early age may put young adolescents at higher risk for poorer sexual and reproductive health outcomes. Most previous studies have identified factors influencing early sexual initiation from a static perspective, and only a few studies have explained the possible reasons for the decline in the prevalence of sexual intercourse. One study indicated that access to formal sexual education may reduce the number of adolescents younger than 15 years who have sexual intercourse [33]. Another study conducted in British Columbia suggested that the presence of protective factors (such as supportive schools and families, opportunities for community and school involvement) in adolescents’ lives may contribute to declines in the proportion of adolescents in grades 7 to 12 who reported ever having sexual intercourse from 1992 to 2003 [34]. There is currently a lack of evidence explaining the change in adolescent sexual intercourse; future research should consider investigating factors that may affect changes in the prevalences of sexual behaviors among young adolescents.

The decreasing prevalence of condom use at last sex over time among young adolescents aged 12 years to 15 years is worrying. Previous studies have suggested that limited access to condoms (eg, restrictive laws or policies that provide contraceptives based on age or marital status) and the sociocultural environment (eg, the stigmatization of condoms) may be the main barriers to condom use among young people [35]. In addition, the decline in condom use at last sex among young adolescents might be explained by the lack of contraceptive knowledge, financial constraints, and the pursuit of sexual pleasure [36]. We found that the prevalence of having multiple sexual partners among adolescents who had ever had sexual intercourse increased over time, which was inconsistent with findings in HICs. The United States (2009-2019) and Canada (1992-2003) reported the prevalence of having multiple sexual partners decreased over time, by 5.2%, among high school students and, by 3.6%, among adolescents in grades 7 to 12 [32,34]. These previous studies conducted in Canada and the United States defined multiple sexual partners as having 3, 4, or more sexual partners during their lifetime, whereas the GSHS defined multiple sexual partners as having 2 or more partners in their lifetime, which may be one reason why the results of this study are inconsistent with previous results.

### Implications and Contribution

Healthy sexual behaviors are an important component of adolescents’ physical and psychological development. This study reported the prevalence and trends of sexual behaviors of young adolescents aged 12 years to 15 years in LMICs, which can provide evidence and important implications for policymakers to implement targeted programs that promote and improve the sexual and reproductive health of adolescents. The prevalences of adolescent sexual behaviors vary widely across countries, and there is an urgent need for the public health sector to develop targeted policy support systems to prevent and reduce risky sexual behaviors among young adolescents in LMICs with high prevalences of risky sexual behaviors. For example, the comprehensive sexuality education (CSE) intervention should be implemented, and its integration into the education system should be encouraged in countries where the prevalence of early sexual intercourse initiation is high or rising. Previous studies have indicated that CSE can increase sexual health knowledge and reduce risky sexual behaviors among young adolescents such as delaying the age of first sexual intercourse or ensuring that the age of sexual initiation is developmentally appropriate for the individual, increasing contraception use, and reducing the number of sexual partners [37,38]. The decline in condom use at last sex reported by some countries was a reminder that policymakers should continue to prioritize potential barriers to condom use among adolescents and explore solutions to these barriers. It is also important to consider gender and age differences among young adolescents when developing strategies to promote adolescent sexual health.

### Limitations

This study also has several limitations. First, the prevalence of “having multiple sexual partners” or “condom use at last sex” among adolescents was based on those who have had sexual intercourse; the sample size of young adolescents who ever had sexual intercourse in some countries was extremely low, which may have caused selection bias and resulted in the lowest or highest prevalences of having multiple sexual partners and condom use in these countries. Therefore, the interpretation of the prevalences of having multiple sexual partners and condom use should be considered within the specific circumstances of the sample area. Second, the sexual behavior information was self-reported, which may have resulted in recall bias or intentional omission of key information. Although the interview questionnaire was anonymous and every effort was made to create a secure environment and maintain confidentiality of the respondents’ information, the prevalences of sexual behaviors may still be underestimated or overestimated. Third, the GSHS was based on surveys of teenagers in school, which did not include those who did not attend school. Previous studies have shown that out-of-school students are more likely to initiate sexual behaviors than in-school students; thus, there may exist differences in sexual-related behaviors between these 2 groups, and the results of this study may not be suitable for generalization to all adolescents. Fourth, due to the data availability for each country, only 17 countries have conducted 2 rounds of surveys on sexual behaviors, and there are differences in the timing and length of surveys between countries. Although this can provide evidence for policy development in each country, our estimates for regional or global trends therefore need to be interpreted with caution. Finally, for some countries where sexual behavior surveys were conducted a long time ago (in the past decade or so), it is necessary to obtain data on recent adolescent sexual behaviors to identify recent trends.

### Conclusions

In conclusion, this study found that the prevalences of sexual behaviors in young adolescents varied by country, region, sex, and age group. Over time, the overall prevalences of ever had sexual intercourse and condom use at last sex decreased, but the prevalence of having multiple sexual partners increased. This study can provide evidence and important implications for policymakers to develop a targeted policy support system to prevent and reduce risky sexual behaviors among young adolescents in LMICs with high prevalences of risky sexual behaviors.

## Appendix

Multimedia Appendix 1 Supplementary file.

## Abbreviations

CDC: Center for Disease Control and Prevention
CSE: comprehensive sexuality education
GSHS: Global School–based Student Health Survey
HIC: high-income country
LMICs: low and middle-income countries
STI: sexually transmitted infection
WHO: World Health Organization
YRBS: Youth Risk Behavior Survey

## Data Availability

Data are available in a public, open access repository. It can be freely accessed via the World Health Organization Noncommunicable Diseases Microdata Repository [19].
