# Supplementary material for: Prevalence and Trends of Sexual Behaviors Among Young Adolescents Aged 12 Years to 15 Years in Low and Middle-Income Countries: Population-Based Study
Source: JMIR Public Health Surveill. 2023 Jun 7;9:e45236. doi: 10.2196/45236 (PMC10285621; doi:10.2196/45236)
Supplement: Multimedia Appendix 1 [file publichealth_v9i1e45236_app1.docx]

**Table S1.** The prevalence of ever had sexual intercourse among young adolescent aged 12 years to 15 years by World Health Organization region, country, sex, and age, 2003-2017.

| **Region/country** | **Total, %** | **Boys, %** | **Girls, %** | **12-13 years, %** | **14-15 years, %** |
| --- | --- | --- | --- | --- | --- |
|  | (95% CI) | (95% CI) | (95% CI) | (95% CI) | (95% CI) |
| **Africa** |  |  |  |  |  |
| Benin | 22.9(17.6-29.4) | 30.1(23.0-38.3) ^*^ | 10.1(6.5-15.4) | 17.9(9.4-31.6) | 24.3(18.6-31.0) |
| Botswana | 16.8(14.3-19.6) | 26.5(22.0-31.6) ^*^ | 9.7(7.5-12.4) | 8.5(5.1-13.8) ^*^ | 18.2(15.3-21.5) |
| Eswatini | 7.4(6.1-8.8) | 12.8(9.9-16.4) ^*^ | 4.1(2.9-5.7) | 4.8(3.3-7.0) | 8.2(6.6-10.2) |
| Ghana | 12.6(9.2-17.1) | 16.7(11.3-23.9) | 8.8(6.2-12.3) | 10.2(4.8-20.2) | 13.9(10.0-19.0) |
| Liberia | 21.0(15.2-28.2) | 28.3(19.1-39.7) | 13.9(9.5-19.8) | 15.3(8.3-26.4) | 22.8(15.9-31.4) |
| Malawi | 8.5(5.7-12.6) | 12.8(8.1-19.6) ^*^ | 4.5(2.5-7.9) | 4.5(2.2-8.9) | 10.3(6.8-15.2) |
| Mauritania | 15.9(11.9-21.0) | 19.3(14.7-24.9) | 11.9 (7.1-19.3) | 14.7(8.9-23.2) | 16.3(12.2-21.3) |
| Mauritius | 9.0(6.9-11.7) | 12.3(8.9-16.7) | 6.7(4.1-10.9) | 3.6(2.0-6.4) ^*^ | 11.7(9.2-14.8) |
| Mozambique | 27.8(19.3-38.1) | 45.2(36.0-54.7) ^*^ | 10.6(4.5-22.7) | 17.9(11.4-27.0) | 31.5(21.4-43.7) |
| Namibia | 25.0(21.7-28.7) | 39.8(34.9-44.9) ^*^ | 15.0(11.8-18.9) | 16.2(11.8-21.8) ^*^ | 28.6(24.8-32.7) |
| Senegal | 22.9(18.3-28.4) | 32.7(25.6-40.7) ^*^ | 8.1(4.8-13.4) | 14.8(11.3-19.2) ^*^ | 27.7(22.1-34.1) |
| Seychelles | 29.2(25.2-33.4) | 31.8(27.1-36.9) | 27.1(22.6-32.1) | 16.8(13.5-20.7) ^*^ | 40.6(35.7-45.6) |
| Sierra Leone | 22.3(16.2-30.0) | 31.1(22.4-41.4) ^*^ | 14.8(9.8-21.7) | 18.5(11.8-27.8) | 24.0(17.8-31.5) |
| Tanzania | 6.8(5.3-8.6) | 12.1(9.2-15.6) ^*^ | 2.4(1.6-3.5) | 5.7(3.8-8.3) | 7.6(5.8-9.8) |
| Uganda | 20.1(17.0-23.7) | 29.0(24.6-33.9) ^*^ | 12.7(10.1-16.0) | 10.3(6.2-16.5) ^*^ | 22.4(19.2-25.8) |
| Zambia | 35.4(27.7-44.0) | 45.9(40.2-51.8) ^*^ | 25.2(15.8-37.8) | 26.9(15.2-42.9) | 40.0(33.2-47.2) |
| Zimbabwe | 12.0(10.3-13.9) | 20.5(17.3-24.0) ^*^ | 5.6(4.3-7.2) | 8.2(6.2-10.8) ^*^ | 12.9(11.0-15.1) |
| **Americas** |  |  |  |  |  |
| Anguilla | 20.5(15.2-27.1) | 34.0(26.1-42.9) ^*^ | 9.2(5.8-14.2) | 9.5(4.5-18.8) ^*^ | 25.4(19.2-32.9) |
| Antigua and Barbuda | 33.2(28.2-38.6) | 45.3(38.6-52.2) ^*^ | 21.4(16.7-26.9) | 23.0(17.3-29.9) ^*^ | 38.6(32.9-44.7) |
| Argentina | 28.9(25.6-32.3) | 34.3(30.0-38.8) ^*^ | 24.3(21.0-28.0) | 13.8(11.0-17.1) ^*^ | 35.9(32.5-39.5) |
| Bahamas | 18.1(15.1-21.6) | 30.4(24.8-36.6) ^*^ | 8.0(6.3-10.3) | 13.8(10.8-17.5) ^*^ | 22.8(18.7-27.5) |
| Barbados | 27.5(23.7-31.7) | 37.2(31.4-43.4) ^*^ | 18.4(14.6-22.8) | 12.5(9.3-16.6) ^*^ | 32.0(27.7-36.6) |
| Belize | 16.4(13.9-19.3) | 25.5(21.5-30.1) ^*^ | 7.8(5.9-10.3) | 9.4(7.0-12.6) ^*^ | 22.9(19.3-26.9) |
| Bolivia | 12.8(10.9-15.0) | 17.4(14.7-20.5) ^*^ | 8.5(6.7-10.9) | 4.4(2.9-6.6) ^*^ | 15.9(13.6-18.5) |
| British Virgin Islands | 24.6(21.9-27.3) | 34.4(29.9-38.9) ^*^ | 16.7(13.7-19.7) | 12.2(9.2-15.2) ^*^ | 35.6(31.6-39.6) |
| Cayman | 28.9(26.0-31.8) | 35.7(31.1-40.3) ^*^ | 22.4(18.7-26.1) | 19.5(15.7-23.3) ^*^ | 35.1(30.9-39.3) |
| Chile | 16.4(13.5-19.9) | 19.7(15.6-24.7) | 13.4(10.3-17.4) | 8.9(6.2-12.7) ^*^ | 21.5(17.6-26.0) |
| Colombia | 22.7(20.0-25.7) | 33.1(29.6-36.9) ^*^ | 15.1(12.4-18.3) | 12.8(10.4-15.7) ^*^ | 29.6(26.6-32.9) |
| Costa Rica | 12.6(11.0-14.5) | 16.0(13.3-19.1) ^*^ | 9.6(8.0-11.5) | 5.8(4.1-8.0) ^*^ | 16.1(14.1-18.4) |
| Curaçao | 13.9(11.2-17.0) | 17.2(13.3-21.9) | 10.9(8.5-14.0) | 4.5(3.0-6.7) ^*^ | 18.0(14.5-22.2) |
| Dominica | 36.1(31.6-40.8) | 46.9(39.9-53.9) ^*^ | 26.8(22.3-31.7) | 22.6(17.8-28.1) ^*^ | 48.5(43.1-54.0) |
| Ecuador | 14.3(12.0-16.9) | 23.0(19.9-26.4) ^*^ | 6.6(5.0-8.5) | 8.7(7.1-10.6) ^*^ | 20.8(17.3-24.9) |
| El Salvador | 13.7(11.1-16.7) | 21.1(17.1-25.6) ^*^ | 6.6(4.8-8.9) | 7.2(4.7-11.0) ^*^ | 16.5(13.4-20.1) |
| Grenada | 25.4(22.0-29.1) | 44.0(37.4-50.7) ^*^ | 13.8(10.7-17.6) | 14.1(10.1-19.2) ^*^ | 34.0(28.8-39.6) |
| Guatemala | 8.3(6.6-10.4) | 14.0(10.8-18.0) ^*^ | 2.8(1.7-4.6) | 4.7(2.7-8.2) | 10.1(7.5-13.6) |
| Guyana | 20.5(16.9-24.5) | 32.3(27.0-38.2) ^*^ | 10.6(7.8-14.2) | 8.8(6.0-12.9) ^*^ | 25.3(21.1-29.9) |
| Honduras | 14.1(12.3-16.2) | 24.3 (20.5-28.6) ^*^ | 6.2(4.9-7.8) | 8.4(6.3-11.1) ^*^ | 19.2(16.2-22.6) |
| Jamaica | 31.9(26.1-38.3) | 55.7(49.0-62.2) ^*^ | 12.6(8.9-17.4) | 24.8(15.9-36.6) | 33.7(27.4-40.7) |
| Paraguay | 14.3(11.9-17.0) | 20.8(17.6-24.3) ^*^ | 8.8(6.7-11.6) | 4.9(3.4-7.0) ^*^ | 19.1(16.1-22.5) |
| Peru | 15.1(13.1-17.4) | 22.3(19.2-25.7) ^*^ | 8.2(6.1-10.9) | 10.0(7.1-13.8) ^*^ | 16.9(14.6-19.5) |
| Saint Kitts and Nevis | 27.5(25.0-30.0) | 42.4(38.0-46.8) ^*^ | 14.8(12.2-17.4) | 16.1(11.9-20.3) ^*^ | 30.5(27.5-33.5) |
| Saint Lucia | 24.1(19.7-29.1) | 36.8(29.7-44.4) ^*^ | 14.9(10.8-20.2) | 15.4(11.0-21.1) ^*^ | 30.2(24.3-36.9) |
| Saint Vincent and the Grenadines | 30.1(24.6-36.3) | 54.2(47.8-60.6) ^*^ | 12.2(8.8-16.7) | 22.9(16.4-31.0) ^*^ | 38.2(31.7-45.2) |
| Suriname | 12.7(9.1-17.4) | 18.0(13.0-24.3) ^*^ | 8.5(5.8-12.2) | 5.3(3.0-9.0) ^*^ | 16.8(12.6-22.2) |
| Trinidad and Tobago | 13.5(10.8-16.7) | 20.0(16.2-24.4) ^*^ | 8.2(5.8-11.3) | 6.9(4.5-10.4) ^*^ | 19.1(15.5-23.2) |
| Uruguay | 23.2(20.8-25.8) | 28.7(26.1-31.4) ^*^ | 18.7(16.0-21.9) | 9.0(6.9-11.6) ^*^ | 28.6(25.8-31.5) |
| Venezuela | 14.0(11.6-16.7) | 27.6(23.9-31.7) ^*^ | 3.4(2.6-4.5) | 9.3(7.4-11.7) ^*^ | 21.5(17.4-26.4) |
| **Eastern Mediterranean** | | |  |  |  |
| Djibouti | 15.0(12.0-18.6) | 22.2(17.9-27.1) ^*^ | 4.8(3.1-7.5) | 10.3(4.5-21.9) | 16.0(13.3-19.0) |
| **European** |  |  |  |  |  |
| Macedonia | 9.0(6.8-12.0) | 14.9(11.0-19.9) ^*^ | 3.1(1.9-5.2) | 5.4(3.2-9.1) | 11.2(8.3-14.9) |
| Tajikistan | 1.2(0.7-2.0) | 2.1(1.2-3.7) ^*^ | 0.1(0.0-0.5) | 0.7(0.3-1.5) | 1.3(0.7-2.3) |
| **Southeast Asia** |  |  |  |  |  |
| Bhutan | 6.2(5.2-7.5) | 12.1(9.8-14.8) ^*^ | 1.6(1.0-2.6) | 5.1(4.0-6.5) | 6.8(5.3-8.6) |
| Indonesia | 0.6(0.4-1.0) | 1.1(0.7-1.7) ^*^ | 032(0.1-0.5) | 0.7(0.4-1.2) | 0.6(0.4-0.9) |
| Nepal | 3.4(2.2-5.1) | 5.3(3.7-7.5) ^*^ | 1.7(0.9-3.4) | 1.6(0.9-3.1) | 4.4(2.8-6.9) |
| Thailand | 5.7(4.4-7.1) | 7.2(5.2-9.8) | 4.4(3.2-6.1) | 2.5(1.5-4.2) ^*^ | 8.1(6.3-10.3) |
| Timor-Leste | 5.7(3.8-8.5) | 7.3(4.3-12.2) | 4.4(2.6-7.3) | 6.9(3.6-12.8) | 5.4(3.5-8.2) |
| **Western Pacific** |  |  |  |  |  |
| Brunei Darussalam | 1.9(1.3-2.9) | 2.1(1.1-4.2) | 1.7(1.0-2.9) | 0.5(0.1-1.9) | 2.5(1.7-3.8) |
| Cambodia | 1.1(0.6-1.9) | 1.1(0.5-2.5) | 1.1(0.6-2.2) | 0.4(0.1-1.7) | 1.4(0.7-2.6) |
| Fiji | 6.7(4.8-9.4) | 11.6(8.4-15.9) ^*^ | 2.8(1.6-4.7) | 1.0(0.2-5.1) ^*^ | 7.3(5.2-10.1) |
| French Polynesia | 23.4(19.7-27.4) | 29.2(24.7-34.2) ^*^ | 18.0(14.1-22.7) | 10.6(8.2-13.5) | 33.9(29.2-38.8) |
| Kiribati | 15.6(13.5-17.9) | 30.0(26.1-34.2) ^*^ | 4.3(3.0-6.0) | 10.6(7.3-15.0) ^*^ | 17.5(15.3-19.9) |
| Lao People's Democratic Republic | 2.5(1.5-4.2) | 4.0(2.1-7.6) | 1.1(0.6-2.3) | 1.1(0.2-6.9) | 2.7(1.6-4.4) |
| Malaysia | 1.2(0.9-1.5) | 1.6(1.2-2.1) ^*^ | 0.8(0.6-1.1) | 0.7(0.4-1.2) | 1..4(1.1-1.9) |
| Mongolia | 4.2(3.3-5.3) | 6.3(4.7-8.3) ^*^ | 2.2(1.7-3.0) | 1.8(1.2-2.7) ^*^ | 6.1(4.6-7.9) |
| Nauru | 18.2(13.1-23.3) | 25.0(15.1-34.9) ^*^ | 13.7(8.1-19.3) | 12.7(5.6-19.8) | 22.3(15.3-29.3) |
| Samoa | 6.7(4.3-10.3) | 12.5(7.8-19.3) | 2.9(1.4-6.2) | 0.9(0.2-3.9) ^*^ | 10.0(6.7-14.6) |
| Tuvalu | 4.6(2.7-6.5) | 9.8(5.6-14.0) | 0.8(0.3-1.9) | 1.9(0.2-3.6) ^*^ | 7.8(4.1-11.5) |
| Vanuatu | 9.9(6.8-14.2) | 17.1(11.6-24.4) ^*^ | 4.1(2.6-6.4) | 5.0(3.0-8.2) | 11.4(7.8-16.4) |
| Viet Nam | 0.8(0.4-1.5) | 0.9(0.4-2.0) | 0.7(0.4-1.5) | 0^*^ | 0.8(0.4-1.5) |
| Wallis and Futuna | 15.6(11.9-20.1) | 24.6(18.9-31.4) ^*^ | 7.9(4.7-13.1) | 14.1(9.9-19.7) | 16.5(12.1-22.2) |

**Notes** Data are prevalence (95% Confidence interval). ^*^ p<0.05 for the difference between sexes, age.

**Table S2.** The prevalence of having multiple sexual partners among young adolescents aged 12 years to 15 years by World Health Organization region, country, sex, and age, 2003-2017.

| **Region/country** | **Total, %** | **Boys, %** | **Girls, %** | **12-13 years, %** | **14-15 years, %** |
| --- | --- | --- | --- | --- | --- |
|  | (95% CI) | (95% CI) | (95% CI) | (95% CI) | (95% CI) |
| **Africa** |  |  |  |  |  |
| Benin | 61.4(53.1-69.1) | 68.6(59.2-76.6) ^*^ | 23.3(13.3-37.6) | 68.8(48.5-83.7) | 60.0(51.2-68.1) |
| Botswana | 52.2(43.1-61.1) | 54.2(44.1-64.0) | 48.1(32.6-64.1) | 60.0(30.8-83.4) | 51.6(43.2-59.8) |
| Eswatini | 57.1(47.7-66.0) | 73.9(58.3-85.2) ^*^ | 25.3(14.0-41.4) | 78.5(49.1-93.2) | 53.0(43.5-62.2) |
| Ghana | 56.4(49.0-63.5) | 59.9(46.5-71.9) | 50.0(34.1-66.0) | 85.7(70.3-93.8) ^*^ | 45.4(35.9-55.3) |
| Liberia | 41.9(30.8-53.9) | 59.2(45.6-71.5) ^*^ | 7.5(2.4-20.9) | 52.6(18.5-84.5) | 39.7(29.4-50.9) |
| Malawi | 36.9(26.2-49.1) | 34.6(22.4-49.3) | 43.0(29.1-58.2) | 32.2(15.4-55.3) | 37.8(25.2-52.3) |
| Mauritania | 60.2(48.9-70.4) | 59.2(45.7-71.4) | 62.0(42.5-78.3) | 51.0(33.2-68.5) | 62.5(50.4-73.1) |
| Mauritius | 52.2(42.0-62.1) | 63.8(52.3-73.9) ^*^ | 37.3(21.3-56.7) | 57.0 (40.3-72.3) | 51.4(39.2-63.4) |
| Mozambique | 48.1(37.6-58.8) | 49.8(40.6-59.0) | 40.8(18.1-68.2) | 42.4(22.2-65.5) | 49.3(33.5-65.3) |
| Namibia | 58.9(54.5-63.1) | 64.5(58.2-70.4) ^*^ | 48.6(40.6-56.7) | 57.0(47.8-65.7) | 59.3(54.4-64.0) |
| Senegal | 59.7(54.6-64.5) | 60.9(54.6-66.8) | 52.1(40.0-63.8) | 59.7(49.5-69.1) | 59.6(50.8-67.9) |
| Seychelles | 63.4(58.6-68.0) | 71.7(64.8-77.7) ^*^ | 55.8(49.1-62.3) | 55.8(47.9-63.5) | 66.3(60.5-71.6) |
| Sierra Leone | 36.2(26.4-47.3) | 42.5(28.4-58.0) | 24.7(14.4-38.9) | 33.8(20.9-49.7) | 36.9(26.9-48.2) |
| Tanzania | 50.4(41.9-58.9) | 51.6(42.2-60.8) | 45.6(29.0-63.1) | 43.2(29.0-58.7) | 54.4(42.7-65.7) |
| Uganda | 51.4(45.0-57.8) | 55.0(47.1-62.6) | 44.6(32.8-57.1) | 33.3(15.5-57.7) | 53.3(46.6-59.9) |
| Zambia | 60.5(54.5-66.1) | 56.6(50.1-63.0) | 67.2(51.2-80.0) | 75.4(60.4-86.1) | 55.1(48.7-61.3) |
| Zimbabwe | 61.2(53.6-68.2) | 62.3(54.1-70.0) | 57.8(46.4-68.5) | 61.0(45.6-74.5) | 61.2(53.4-68.4) |
| **Americas** |  |  |  |  |  |
| Anguilla | 60.9(48.9-71.6) | 70.8(58.3-80.8) ^*^ | 30.1(15.6-50.0) | 47.3(12.8-84.5) | 63.1(51.2-73.6) |
| Antigua and Barbuda | 68.6(63.8-73.0) | 73.6(68.6-78.1) ^*^ | 58.2(48.7-67.2) | 68.1(59.3-75.8) | 68.7(62.8-74.1) |
| Argentina | 55.5(53.2-57.7) | 61.2(57.9-64.4) ^*^ | 48.7(45.5-52.1) | 55.6(47.1-63.7) | 55.5(53.1-57.8) |
| Bahamas | 61.3(54.0-68.2) | 66.9(59.8-73.4) ^*^ | 44.0(30.3-58.6) | 58.5(46.2-69.9) | 63.2(55.6-70.2) |
| Barbados | 59.5(53.3-65.4) | 63.3(55.1-70.8) | 52.4(42.4-62.2) | 51.4(33.7-68.8) | 60.5(54.2-66.4) |
| Belize | 64.3(57.5-70.6) | 72.3(65.8-78.1) ^*^ | 39.9(27.4-53.8) | 70.6(57.5-81.0) | 61.9(53.4-69.6) |
| Bolivia | 46.4(40.2-52.7) | 51.9(44.9-58.8) | 36.1(25.2-48.6) | 58.4(38.6-75.8) | 45.1(39.0-51.4) |
| British Virgin Islands | 66.0(60.1-71.9) | 73.7(66.6-80.8) ^*^ | 53.4(43.4-63.4) | 66.1(53.7-78.5) | 65.9(59.1-72.7) |
| Cayman | 63.3(57.4-69.2) | 66.9(59.4-74.4) | 58.0(48.5-67.5) | 54.5(43.5-65.5) | 66.6(59.6-73.6) |
| Chile | 46.2(37.1-55.6) | 56.6(46.0-66.7) ^*^ | 32.3(21.5-45.3) | 33.1(20.9-48.2) | 49.8(38.2-61.5) |
| Colombia | 52.5(48.3-56.6) | 64.8(60.4-69.0) ^*^ | 32.8(25.3-41.3) | 48.6(41.9-55.3) | 53.7(48.2-59.0) |
| Costa Rica | 50.7(43.5-57.9) | 56.8(47.5-65.6) | 41.6(32.0-51.8) | 45.7(28.2-64.4) | 51.6(44.2-59.0) |
| Curaçao | 39.2(33.3-45.5) | 42.6(33.3-52.4) | 34.6(25.9-44.4) | 30.4(16.7-48.8) | 40.2(34.0-46.8) |
| Dominica | 67.3(62.6-71.7) | 72.9(66.7-78.3) ^*^ | 58.9(51.0-66.2) | 58.7(50.4-66.5) | 71.0(65.6-75.8) |
| Ecuador | 48.7(45.2-52.3) | 54.1(50.8-57.3) ^*^ | 32.3(25.6-39.7) | 47.5(41.0-54.2) | 49.3(43.6-55.0) |
| El Salvador | 52.4(46.8-58.0) | 58.5(52.0-64.8) ^*^ | 33.3(20.4-49.2) | 54.3(40.0-68.0) | 52.0(46.0-58.0) |
| Grenada | 60.4(55.0-65.6) | 63.8(56.7-70.3) | 53.6(43.8-63.2) | 46.6(37.0-56.4) ^*^ | 64.7(57.9-71.0) |
| Guatemala | 68.3(56.4-78.2) | 77.1(59.2-88.6) ^*^ | 26.2(8.1-58.9) | 35.3(22.3-50.9) ^*^ | 76.3(66.2-84.0) |
| Guyana | 58.4(52.6-63.9) | 64.0(56.0-71.4) ^*^ | 43.9(36.8-51.4) | 51.4(36.8-65.9) | 59.4(53.5-65.1) |
| Honduras | 45.8(38.9-53.0) | 46.8(38.7-55.0) | 42.9(30.6-56.2) | 41.0(27.6-55.8) | 47.7(41.5-54.0) |
| Jamaica | 70.4(64.0-76.1) | 75.9(69.1-81.6) ^*^ | 50.7(37.1-64.2) | 72.1(51.7-86.1) | 70.1(64.0-75.6) |
| Paraguay | 50.4(45.6-55.1) | 60.9(53.5-67.9) ^*^ | 29.6(21.6-39.1) | 52.1(32.2-71.4) | 50.1(44.6-55.7) |
| Peru | 47.4(43.1-51.9) | 55.8(50.4-61.1) ^*^ | 25.3(16.7-36.4) | 38.5(25.8-53.1) | 49.3(43.9-54.6) |
| Saint Kitts and Nevis | 67.7(62.5-72.9) | 71.5(65.2-77.8) | 58.4(49.0-67.8) | 78.1(65.7-90.5) | 66.3(60.6-72.0) |
| Saint Lucia | 68.1(61.6-73.9) | 80.1(72.2-86.2) ^*^ | 46.4(37.2-55.8) | 71.2(55.2-83.2) | 67.0(59.4-73.8) |
| Saint Vincent and the Grenadines | 67.5(61.5-72.9) | 72.9(66.3-78.6) ^*^ | 49.7(38.4-61.0) | 64.1(53.9-73.1) | 69.7(62.5-76.1) |
| Suriname | 51.3(45.2-57.4) | 60.1(52.1-67.7) ^*^ | 36.5(25.3-49.3) | 52.7(31.0-73.4) | 51.1(44.3-57.7) |
| Trinidad and Tobago | 59.9(51.8-67.5) | 70.8(62.2-78.1) ^*^ | 38.0(27.2-50.3) | 55.7 (40.7-69.7) | 61.2(52.6-69.1) |
| Uruguay | 48.0(44.9-51.2) | 57.6(52.2-62.9) ^*^ | 36.1(30.3-42.3) | 39.4(28.1-52.0) | 49.1(46.2-51.9) |
| Venezuela | 49.6(42.5-56.7) | 52.9(45.1-60.5) | 29.1(15.0-48.8) | 48.8(39.8-57.8) | 50.1(43.3-57.0) |
| **Eastern Mediterranean** | |  |  |  |  |
| Djibouti | 71.2(61.1-79.5) | 72.3(62.2-80.5) | 63.9(37.4-83.9) | 63.2(39.1-82.2) | 72.2(62.1-80.4) |
| **European** |  |  |  |  |  |
| Macedonia | 38.7(30.8-47.4) | 43.0(33.3-53.3) | 18.4(8.8-34.6) | 43.5(28.3-59.9) | 37.4(28.5-47.2) |
| Tajikistan | 45.9(38.4-53.6) | 48.4(40.9-55.9) ^*^ | 0(0-0) | 29.3(18.4-43.3) | 47.9(40.3-55.6) |
| **Southeast Asia** | | |  |  |  |
| Bhutan | 53.2(46.2-60.1) | 55.7(48.2-63.0) | 38.5(22.1-58.0) | 44.3(32.5-56.8) | 56.4(48.0-64.4) |
| Indonesia | 54.4(40.0-68.2) | 52.5(36.2-68.3) | 61.2(33.0-83.4) | 57.0(40.1-72.4) | 51.6(30.0-72.5) |
| Nepal | 58.5(45.7-70.2) | 57.9(43.6-71.1) | 59.9(35.9-79.9) | 65.8(37.1-86.2) | 56.8(43.9-68.9) |
| Thailand | 55.9(44.6-66.6) | 66.6(51.6-78.8) | 41.4 (29.2-54.7) | 28.8(12.6-53.2) | 62.4(50.5-72.9) |
| Timor-Leste | 55.4(37.8-71.8) | 66.9(41.1-85.4) | 39.9(20.8-62.6) | 46.5(17.2-78.5) | 59.0(41.2-74.8) |
| **Western Pacific** |  |  |  |  |  |
| Brunei Darussalam | 40.6(25.4-57.9) | NA | NA | NA | NA |
| Cambodia | 45.2(32.0-59.1) | NA | NA | NA | NA |
| Fiji | 51.0(35.1-66.6) | 59.6(48.7-69.5) | 48.3(30.8-66.3) | NA | NA |
| French Polynesia | 47.5(42.0-53.1) | 57.7(51.1-64.1) ^*^ | 32.5(26.0-39.7) | 53.9(39.5-67.6) | 45.9(40.9-51.0) |
| Kiribati | 54.4(47.1-61.6) | 56.9(48.7-64.7) | 40.6(26.9-56.0) | 39.6(25.9-55.2) | 57.8(49.8-65.5) |
| Lao People's Democratic Republic | 59.6(41.0-75.8) | NA | NA | NA | NA |
| Malaysia | 53.0(46.9-58.9) | 57.6(47.5-67.1) | 44.6(35.2-54.3) | 56.8(41.5-70.9) | 52.1(44.6-59.4) |
| Mongolia | 49.8(42.0-57.7) | 55.6(47.9-63.1) | 34.8(20.8-51.9) | 46.4(30.0-63.5) | 50.7(41.3-60.0) |
| Nauru | 73.7(60.2-87.2) | NA | NA | NA | NA |
| Samoa | 67.5(62.4-72.1) | NA | NA | NA | NA |
| Tuvalu | 62.2(41.5-82.9) | NA | NA | NA | NA |
| Vanuatu | 56.9(49.6-64.0) | 67.5(55.3-77.8) ^*^ | 21.7(9.8-41.4) | 21.7(5.5-57.0) | 61.8(50.3-72.1) |
| Viet Nam | 7.4(0.9-40.4) | NA | NA | NA | NA |
| Wallis and Futuna | 59.5(48.5-69.7) | 64.7(50.2-76.9) | 45.9(29.3-63.5) | 57.9(44.3-70.4) | 60.4(44.4-74.5) |

**Notes** Data are prevalence (95% Confidence interval). ^*^ p<0.05 for the difference between sexes, age. NA: Not Applicable.

**Table S3.** The prevalence of condom use at last sex among young adolescents aged 12 years to 15 years by WHO region, country, sex, and age, 2003-2017.

| **Region/country** | **Total, %** | **Boys, %** | **Girls, %** | **12-13 years, %** | **14-15 years, %** |
| --- | --- | --- | --- | --- | --- |
|  | (95% CI) | (95% CI) | (95% CI) | (95% CI) | (95% CI) |
| **Africa** |  |  |  |  |  |
| Benin | 33.9(23.3-46.4) | 28.7(18.0-42.5) ^*^ | 61.3(46.4-74.4) | 40.7(19.5-66.0) | 32.5(22.2-44.8) |
| Botswana | 55.7(50.9-60.5) | 51.5(46.8-56.2) | 64.1(47.4-78.0) | 32.4(16.7-53.4) | 57.6(52.0-62.9) |
| Eswatini | 56.2(48.8-63.4) | 56.2(45.6-66.2) | 56.3(41.2-70.4) | 32.6(13.4-60.2) | 60.8(51.8-69.1) |
| Ghana | 41.9(28.2-57.0) | 34.7(25.5-45.2) | 55.2(30.2-77.8) | 44.6(22.0-69.8) | 40.9(28.1-55.0) |
| Liberia | 48.6(35.2-62.1) | 41.4(24.6-60.5) | 62.7(42.4-76.4) | 40.7(15.4-72.1) | 50.2(35.0-65.4) |
| Malawi | 60.1(48.7-70.5) | 56.9(44.1-68.8) | 68.7(46.7-84.6) | 27.7(12.0-51.8) ^*^ | 66.3(53.7-77.0) |
| Mauritania | 55.5(44.3-66.2) | 56.9(44.9-68.2) | 52.8(35.2-69.8) | 71.8(51.2-86.0) | 51.4(42.1-60.6) |
| Mauritius | 50.2(39.3-61.0) | 58.0(47.3-67.9) | 40.2(28.0-53.9) | 35.9(19.8-56.0) | 52.4(40.4-64.1) |
| Mozambique | 72.7(60.5-82.2) | 69.2(55.5-80.2) | 87.3(78.6-92.8) | 48.9(21.9-76.5) | 77.8(66.7-86.0) |
| Namibia | 68.9(61.6-75.4) | 66.5(58.7-73.5) | 73.1(61.5-82.3) | 49.2(40.6-57.9) ^*^ | 73.4(66.1-79.6) |
| Senegal | 52.6(41.5-63.4) | 50.8(38.7-62.9) | 63.2(54.7-71.0) | 38.4(25.5-53.2) | 57.0(44.7-68.5) |
| Seychelles | 51.2(46.9-55.4) | 52.9(45.6-60.0) | 49.6(43.0-56.2) | 55.3(48.0-62.3) | 49.6(44.6-54.8) |
| Sierra Leone | 40.5(29.4-52.7) | 48.6(33.6-63.9) | 25.8(17.2-36.9) | 39.9(26.4-55.2) | 40.7(28.5-54.2) |
| Tanzania | 28.7(22.6-35.8) | 29.4(22.1-38.1) | 25.7(14.4-41.5) | 30.5(18.8-45.5) | 27.7(22.1-34.1) |
| Uganda | 58.6(48.2-68.4) | 59.4(47.4-70.5) | 57.1(40.7-72.0) | 52.4(30.9-73.0) | 59.3(48.7-69.0) |
| Zambia | 51.9(42.0-61.6) | 54.5(41.7-66.8) | 47.3(33.7-61.2) | 29.5(13.2-53.6) | 59.9(47.7-71.0) |
| Zimbabwe | 46.6(40.5-52.7) | 46.2(39.2-53.3) | 47.6(35.5-59.9) | 54.2(37.8-69.7) | 45.4(38.5-52.5) |
| **Americas** |  |  |  |  |  |
| Anguilla | 64.2(54.9-72.6) | 64.0(50.0-76.0) | 65.0(42.5-82.3) | 64.3(38.6-83.8) | 64.2(54.1-73.2) |
| Antigua and Barbuda | 68.7(62.8-74.1) | 68.2(61.2-74.5) | 69.7(60.0-77.9) | 56.4(42.5-69.5) | 72.5(65.8-78.4) |
| Argentina | 75.7(71.2-79.7) | 77.4(71.5-82.5) | 73.7(68.0-78.6) | 73.0(63.1-81.0) | 76.2(71.6-80.3) |
| Bahamas | 59.8(49.7-69.1) | 56.0(43.4-67.9) | 71.5(61.2-79.9) | 52.8(39.5-65.8) | 64.4(50.8-76.0) |
| Barbados | 64.8(59.5-69.8) | 64.3(57.0-71.0) | 65.7(57.2-73.3) | 58.9(42.0-73.9) | 65.5(60.0-70.6) |
| Belize | 66.5(59.6-72.7) | 67.3(60.1-73.8) | 63.8(52.6-73.8) | 65.5(52.7-76.3) | 66.9(58.0-74.6) |
| Bolivia | 64.7(58.4-70.5) | 69.6(62.4-76.0) | 55.5(43.9-66.5) | 63.6(41.8-80.9) | 64.8(58.0-71.1) |
| British Virgin Islands | 71.9(66.3-77.5) | 75.0(68.0-82.0) | 67.0(57.6-76.4) | 72.4(60.7-84.1) | 71.8(65.4-78.2) |
| Cayman | 69.9(64.3-75.5) | 72.9(65.8-80.0) | 65.5(56.4-74.6) | 75.6(66.1-85.1) | 67.9(61.0-74.8) |
| Chile | 53.7(45.7-61.5) | 50.8(40.1-61.5) | 57.6(45.4-68.9) | 52.1(38.2-65.7) | 54.1(44.1-63.8) |
| Colombia | 53.9(49.7-58.1) | 58.0(53.7-62.2) | 47.4(39.3-55.6) | 49.9(44.1-55.7) | 55.1(49.8-60.4) |
| Costa Rica | 62.9(57.5-67.9) | 61.6(52.5-70.0) | 64.7(55.1-73.3) | 57.1(44.3-69.0) | 63.9(58.3-69.2) |
| Curaçao | 62.1(54.7-68.9) | 71.8(62.5-79.5) ^*^ | 48.6(37.0-60.3) | 48.8(25.2-72.9) | 63.5(55.8-70.6) |
| Dominica | 64.4(58.9-69.7) | 61.6(53.0-69.5) | 68.8(61.8-75.0) | 54.4(44.6-63.9) | 68.7(62.7-74.2) |
| Ecuador | 46.2(41.5-50.9) | 50.2(45.2-55.2) ^*^ | 33.7(25.6-42.9) | 41.2(34.1-48.6) | 48.7(43.1-54.3) |
| El Salvador | 73.8(65.7-80.5) | 76.5(67.1-84.0) | 65.2(50.3-77.7) | 71.5(50.2-86.2) | 74.2(66.0-81.1) |
| Grenada | 55.1(49.4-60.6) | 56.0(49.5-62.2) | 53.3(40.0-66.1) | 49.8(37.5-62.2) | 56.7(48.8-64.3) |
| Guatemala | 58.5(40.4-74.6) | 61.3(44.2-76.0) | 45.1(20.6-72.2) | 43.0(15.0-76.4) | 62.3(41.4-79.4) |
| Guyana | 61.4(56.7-65.9) | 59.8(55.1-64.4) | 65.4(52.4-76.4) | 61.3(48.0-73.1) | 61.4(57.0-65.6) |
| Honduras | 62.3(54.4-69.6) | 60.8 (51.5-69.3) | 67.1(55.7-76.7) | 62.0 (46.3-75.6) | 62.4(54.4-69.8) |
| Jamaica | 65.6(58.8-71.8) | 66.4(59.8-72.3) | 62.8(48.6-75.1) | 65.5 (55.4-74.3) | 65.6(58.1-72.4) |
| Paraguay | 73.4(67.1-78.8) | 77.3(69.0-83.8) | 65.7(57.1-73.4) | 75.7(57.3-87.9) | 73.1(66.8-78.5) |
| Peru | 63.5(58.3-68.4) | 63.8(58.5-68.7) | 62.9(53.4-71.6) | 65.7(53.8-76.0) | 63.1(57.2-68.6) |
| Saint Kitts and Nevis | 53.0(47.4-58.6) | 49.9(43.0-56.8) | 60.5(51.1-69.9) | 51.0(36.1-65.9) | 53.2(47.2-59.2) |
| Saint Lucia | 51.5(43.2-59.8) | 46.4(37.1-55.9) | 60.9(49.9-70.9) | 37.7(26.2-50.8) | 56.4(46.1-66.2) |
| Saint Vincent and the Grenadines | 58.4(52.5-64.1) | 56.9(49.8-63.7) | 63.5(53.9-72.1) | 54.1(43.1-64.6) | 61.3(55.1-67.2) |
| Suriname | 64.5(55.6-72.5) | 70.0(57.1-80.4) | 55.2(46.0-64.1) | 49.0(28.6-69.6) | 67.2(59.4-74.3) |
| Trinidad and Tobago | 54.1(47.5-60.6) | 57.7(49.7-65.4) | 46.8(35.5-58.5) | 50.0(38.8-61.2) | 55.3(47.9-62.6) |
| Uruguay | 85.1(82.1-87.6) | 87.4(83.6-90.5) | 82.1(76.1-86.9) | 82.4(67.9-91.2) | 85.4(82.6-87.8) |
| Venezuela | 54.9(47.8-61.8) | 55.6(47.6-63.3) | 50.0(39.4-60.7) | 48.5(41.4-55.7) | 59.3(50.9-67.3) |
| **Eastern Mediterranean** | | |  |  |  |
| Djibouti | 64.1(51.6-75.0) | 64.9(51.4-76.3) | 59.4(31.5-82.3) | 64.6(40.9-82.7) | 64.1(51.0-75.4) |
| **European** |  |  |  |  |  |
| Macedonia | 77.7(68.1-85.0) | 76.6(65.9-84.7) | 83.0(68.3-91.6) | 80.7(58.7-92.4) | 76.8(65.6-85.2) |
| Tajikistan | 57.3(52.2-62.2) | 60.4(55.4-65.2) ^*^ | 0.0(0.0-0.0) | 35.0(17.3-58.1) | 60.1(56.1-63.9) |
| **Southeast Asia** |  |  |  |  |  |
| Bhutan | 52.5(44.5-60.4) | 53.2(45.3-60.9) | 48.6(29.4-68.2) | 62.2(45.5-76.5) | 49.1(39.8-58.3) |
| Indonesia | 31.1(20.1-44.9) | 29.7(16.5-47.5) | 36.1(14.1-65.9)) | 37.8(22.7-55.6) ^*^ | 23.6(9.6-47.2)) |
| Nepal | 52.6(40.6-64.4) | 57.8(42.7-71.6) | 39.1(19.5-62.9) | 55.9(27.3-81.0) | 51.9(37.7-65.9) |
| Thailand | 66.8(58.8-74.0) | 73.3(60.4-83.2) | 58.0(47.6-67.7) | 78.6(61.2-89.5) | 64.0(54.6-72.4) |
| Timor-Leste | 47.0(32.9-61.6) | 47.6(28.4-67.5) | 46.2(26.4-67.2) | 50.2(25.5-74.8) | 45.6(29.5-62.7) |
| **Western Pacific** |  |  |  |  |  |
| Brunei Darussalam | 35.0(21.1-52.0) | NA | NA | NA | NA |
| Cambodia | 75.3(59.4-86.4) | NA | NA | NA | NA |
| Fiji | 49.7(38.4-61.0) | 52.9((40.5-64.9) | 38.8(23.0-57.4) | 43.2(15.3-76.2) | 49.1(37.9-60.3) |
| French Polynesia | 57.7(54.3-61.1) | 62.2(58.2-66.0) | 51.2(42.9-59.5) | 67.9(58.1-76.4) | 55.1(50.7-59.4) |
| Kiribati | 20.5(15.1-27.3) | 21.0(15.2-28.2) | 17.9(7.8-35.9) | 34.7(19.4-54.0) | 17.2(12.7-22.9) |
| Lao People's Democratic Republic | 60.6(39.6-78.3) | NA | NA | NA | NA |
| Malaysia | 37.4(29.4-46.2) | 45.0(37.7-52.6) | 23.7(12.6-40.1) | 38.7(24.2-55.6) | 37.2(28.7-46.5) |
| Mongolia | 48.2(39.9-56.6) | 53.2(42.9-63.3) | 35.2(21.3-52.2) | 44.0(25.8-64.1) | 49.2(40.9-57.6) |
| Nauru | 51.8(36.5-67.1) | NA | NA | NA | NA |
| Samoa | 46.7(30.7-63.5) | NA | NA | NA | NA |
| Tuvalu | 56.9(35.7-78.1) | NA | NA | NA | NA |
| Vanuatu | 55.2(46.7-63.5) | 58.9(47.5-69.4) | 43.0(28.9-58.3) | 51.6(21.0-81.0) | 55.7(46.1-64.9) |
| Viet Nam | 46.7(18.3-77.4) | NA | NA | NA | NA |
| Wallis and Futuna | 43.5(32.4-55.3) | 53.1(41.3-64.7) ^*^ | 18.0(6.7-40.2) | 43.3(26.8-61.5) | 43.6(32.0-56.0) |

**Notes** Data are prevalence (95% Confidence interval). ^*^ p<0.05 for the difference between sexes, age. NA: Not Applicable.

**Table S4.** The trends in the prevalence of ever had sexual intercourse among young adolescent aged 12 years to 15 years, between the earliest and latest surveys, by WHO region, country, sex, and age.

| **Region/country Survey Year** | | **Total, %** | **Boys, %** | **Girls, %** | **12-13 years, %** | **14-15 years, %** |  |
| --- | --- | --- | --- | --- | --- | --- | --- |
|  |  | (95% CI) | (95% CI) | (95% CI) | (95% CI) | (95% CI) |  |
| **Africa** |  |  |  |  |  |  |  |
| Eswatini | 2003-2013 | -3.0(-4.8 to -1.2)* | -6.4(-10.3 to -2.5)* | -2.3(-4.0 to -0.6)* | -1.8(-4.7 to -1.1)* | -3.3(-5.5 to -1.1)* |  |
| Ghana | 2007-2012 | -3.3(-5.9 to -0.7)* | -2.3(-6.5 to 1.9) | -3.8(-7.0 to -0.6)* | -4.7(-9.1 to -0.3)* | -2.4(-5.7 to 0.9) |  |
| Mauritius | 2011-2017 | -12.6(-14.9 to -10.3)* | -16.2(-20.0 to -12.4)* | -8.3(-11.0 to -5.6)* | -5.0 (-7.6 to -2.4) | -18.2(-21.4 to -15.0)* |  |
| Namibia | 2004-2013 | 2.0(-0.9 to 4.9) | 4.8(-0.4 to 10.0) | -0.2(-3.3 to 2.9) | -2.8(-7.5 to 1.9) | 4.3(0.8 to 7.8)* |  |
| Seychelles | 2007-2015 | 7.9(4.3 to 11.5)* | 1.5(-4.5 to 7.5) | 0(-4.9 to 4.9) | 2.6(-1.7 to 6.9) | 13.4(8.0 to 18.8)* |  |
| **Americas** |  |  |  |  |  |  | |
| Anguilla | 2009-2016 | -2.5(-7.6 to 2.6) | 1.6(-7.3 to 10.5) | -6.1(-11.5 to -0.7)* | -1.7(-8.0 to 4.6) | -5.7(-12.8 to 1.4) |  |
| Argentina | 2007-2012 | 2.0(-0.5 to 4.5) | -3.0(-7.1 to 1.1) | 5.2(2.2 to 8.2)* | 4.0(0.5 to 7.5)* | 3.7(0.7 to 6.7)* |  |
| Guatemala | 2009-2015 | 0.4(-0.9 to 1.7) | 2.2(-0.2 to 4.6) | -1.1(-2.2 to 0.1) | 0.9(-0.9 to 2.7) | 0.2(-1.5 to 1.9) |  |
| Guyana | 2004-2010 | -0.8(-4.2 to 2.6) | -3.2(-9.6 to 3.2) | 0.5(-2.8 to 3.8) | -4.8(-9.9 to 0.3) | 0.8(-3.5 to 5.1) |  |
| Suriname | 2009-2016 | -5.3(-8.4 to -2.2) * | -7.7(-12.9 to -2.5)* | -3.3(-6.8 to 0.2) | -2.5(-6.2 to 1.2) | -5.8(-9.9 to -1.7)* |  |
| Trinidad and Tobago | 2011-2017 | -4.1(-6.3 to -1.9) * | -3.3(-6.9 to 0.3) | -4.2(-6.8 to -1.6)* | 0.2(-2.0 to 2.4) | -7.7(-11.0 to -4.1)* |  |
| Uruguay | 2006-2012 | 1.1(-1.2 to 3.4) | -3.9(-7.7 to -0.1)* | 3.9(1.2 to 6.6)* | -1.0(-3.8 to 1.8) | -1.8(-5.0 to 1.4) |  |
| **Southeast Asia** |  |  |  |  |  |  |  |
| Indonesia | 2007-2015 | 0.2(-0.1 to 0.5) | 0.6(0.1 to 1.1)* | 0.1(-0.2 to 0.4) | 0.4(0.1 to 0.8)* | 0.2(-0.2 to 0.6) |  |
| Thailand | 2008-2015 | 0.1(-1.2 to 1.4) | 0.5(-1.5 to 2.5) | -0.3(-1.9 to 1.3) | -0.2(-1.5 to 1.0) | 0(-0.2 to 2.2) |  |
| **Western Pacific** |  |  |  |  |  |  |  |
| Fiji | 2010-2016 | -3.0(-5.2 to 0.8) | -6.0(-10.4 to -1.6) * | -0.6(-2.4 to 1.2) | -3.0(-6.0 to 0.1) | -4.9(-7.5 to -2.3)* |  |
| Samoa | 2011-2017 | -24.6(-28.6 to -20.6)* | -36.2(-44.2 to -28.2)* | -16.9(-20.9 to -12.9)* | -21.2(-27.0 to -15.4)* | -24.5(-29.5 to -19.5)* |  |
| Vanuatu | 2011-2016 | 1.0(-1.8 to 3.8) | 5.7(0.4 to 11.0) ^*^ | -2.4(-5.2 to 0.4) | -0.3(-3.9 to 3.3) | -1.3(-5.3 to 2.7) |  |

**Notes** Data are prevalence (95% Confidence interval). * p<0.05, represent significant downward or upward trends in prevalence.

**Table S5.** The trends in the prevalence of having multiple sexual partners among young adolescent aged 12 years to 15 years, between the earliest and latest surveys, by WHO region, country, sex and age.

| **Region/country** | **Survey Year** | **Total, %** | **Boys, %** | **Girls, %** | **12-13 years, %** | **14-15 years, %** |
| --- | --- | --- | --- | --- | --- | --- |
|  |  | (95% CI) | (95% CI) | (95% CI) | (95% CI) | (95% CI) |
| **Africa** |  |  |  |  |  |  |
| Eswatini | 2003-2013 | 5.3(-6.6 to 17.2) | 15.8(2.4 to 29.2) * | -18.0(-35.8 to -0.2)* | 13.6(-11.4 to 38.6) | 3.3(-9.8 to 16.4) |
| Ghana | 2007-2012 | -3.5(-14.1 to 7.1) | -4.3(-17.4 to 8.8) | -3.3(-20.8 to 14.2) | 29.0(12.7 to 45.3)* | -16.0(-28.2 to -3.8)* |
| Mauritius | 2011-2017 | 1.4(-8.3 to 11.1) | 5.6(-6.5 to 17.7) | 0.1(-14.8 to 15.0) | 27.2 (1.7 to 52.7)* | -3.1(-13.6 to 7.4) |
| Namibia | 2004-2013 | 14.2(7.3 to 21.1)* | 15.3(6.6 to 24.0)* | 10.6(-0.6 to 21.8) | 13.7(-1.6 to 29.0) | 14.2(6.5 to 21.9)* |
| Seychelles | 2007-2015 | 10.5(1.9 to 19.1)* | 13.3(2.1 to 24.5)* | 12.1(-1.0 to 25.2) | 8.6(-6.9 to 24.1) | 10.9(0.6 to 21.2)* |
| **Americas** |  |  |  |  |  |  |
| Anguilla | 2009-2016 | -2.5(-15.8 to 10.8) | 2.8(-12.4 to 18.0) | -25.5(-49.1 to -1.9) | -1.6(-34.3 to 31.1) | -3.9(-18.3 to 10.5) |
| Argentina | 2007-2012 | 0.4(-5.1 to 5.9) | -3.0(-9.4 to 3.7) | 7.2(-1.5 to 15.9) | 5.3(-13.0 to 23.6) | 0(-5.7 to 5.7) |
| Guatemala | 2009-2015 | 11.6(4.0 to 19.2)* | 13.1(4.7 to 21.5)* | -7.5(-21.6 to 6.6) | -24.1(-43.2 to -5.0)* | 20.1(12.1 to 28.1)* |
| Guyana | 2004-2010 | -3.9(-13.2 to 5.4) | -8.3(-18.9 to 2.3) | 9.3(-6.9 to 25.5) | -13.7(-37.7 to 10.3) ^*^ | -2.3(-12.4 to 7.8) |
| Suriname | 2009-2016 | 0.1(-10.8 to 11.0) | 0.7(-12.8 to 14.2) | -0.8(-18.2 to 16.6) | -9.1(-37.9 to 19.7) | 1.5(-10.3 to 13.3) |
| Trinidad and Tobago | 2011-2017 | -0.3(-7.6 to 7.0) | 2.9(-5.5 to 11.3) | -9.1(-21.5 to 3.3) | 7.5(-8.5 to 23.5) | -1.5(-9.6 to 6.6) |
| Uruguay | 2006-2012 | -2.0(-8.0 to 4.0) | -3.2(-10.8 to 4.4) | 5.1(-3.8 to 14.0) | -11.2(-27.5 to 4.6) | -0.8(-7.4 to 5.8) |
| **Southeast Asia** |  |  |  |  |  |  |
| Indonesia | NA | NA | NA | NA | NA | NA |
| Thailand | 2008-2015 | 7.4(-3.9 to 18.7) | 4.4(-10.1 to 18.9) | 8.2(-8.1 to 24.5) | -22.9(-45.8 to 0.1) | 14.9(2.2 to 27.6)* |
| **Western Pacific** |  |  |  |  |  |  |
| Fiji | 2010-2016 | 0(-14.4 to 14.4) | 5.0(-11.2 to 21.2) | 12.6(-17.4 to 42.6) | 43.5(15.4 to 71.6) | 6.3(-8.5 to 21.1) |
| Samoa | 2011-2017 | 0(-15.6 to 15.6) | -19.5(-41.0 to 2.0) | 10.9(-36.5 to 14.7) | 12.1(-57.4 to 81.6) | 4.1(-13.2 to 21.4) |
| Vanuatu | 2011-2016 | 15.5(-0.3 to 31.3) | 14.0(-5.7 to 33.7) | 0.7(-21.0 to 22.4) | -26.5(-60.1 to .7.1) | -9.5(-26.2 to 7.2) |

**Notes** Data are prevalence (95% Confidence interval). * p<0.05, represent significant downward or upward trends in prevalence.NA: Not Applicable.

**Table S6.** The trends in the prevalence of condom use at last sex among young adolescent aged 12 years to 15 years, between the earliest and latest surveys, by WHO region, country, sex, and age.

| **Region/country** | **Survey Year** | **Total, %** | **Boys, %** | **Girls, %** | **12-13 years, %** | **14-15 years, %** |
| --- | --- | --- | --- | --- | --- | --- |
|  |  | (95% CI) | (95% CI) | (95% CI) | (95% CI) | (95% CI) |
| **Africa** |  |  |  |  |  |  |
| Eswatini | 2003-2013 | 12.7(0.8 to 24.6)* | 15.6(0.8 to 30.4)* | 8.8(-11.1 to 28.7) | 4.4(-23.2 to 32.0) | 14.8(2.0 to 27.6)* |
| Ghana | 2007-2012 | -9.7(-20.3 to 0.9) | -13.3(-26.3 to -0.3)* | -1.8(-19.2 to 15.6) | -16.5(-37.7 to 4.7) | -6.3(-18.5 to 5.9) |
| Mauritius | 2011-2017 | 6.8(-2.9 to 16.5) | 12.6(0.2 to 25.0)* | 0.5(-14.6 to 15.6) | 0.2 (-24.9 to 25.3) | 7.6(-2.9 to 18.1) |
| Namibia | 2004-2013 | 14.6(7.9 to 21.3)* | 9.5(0.9 to 18.1)* | 22.9(12.3 to 33.5)* | 6.6(-8.8 to 22.0) | 16.2(8.9 to 23.5)* |
| Seychelles | 2007-2015 | -2.5(-11.2 to 6.2) | -2.2(-13.8 to 9.4) | -1.9(-15.1 to 11.3) | -3.5(-18.9 to 11.9) | -1.9(-12.4 to 8.6) |
| **Americas** |  |  |  |  |  |  |
| Anguilla | 2009-2016 | -4.8(-17.7 to 8.1) | -11.6(-26.7 to 3.5) | 7.2(-17.0 to 31.4) | 0.5(-30.9 to 31.9) | -6.1(-20.3 to 8.1) |
| Argentina | 2007-2012 | -3.1(-7.6 to 1.4) | -4.2(-9.7 to 1.3) | -1.1(-8.7 to 6.5) | -11.4(-24.8 to 20.0) | -2.1(-6.8 to 2.6) |
| Guatemala | 2009-2015 | 1.4(-6.5 to 9.3) | 0.9(-8.2 to 10.0) | -1.5(-17.0 to 14.0) | -11.7(-31.3 to 7.9) | 4.8(-3.7 to 13.3) |
| Guyana | 2004-2010 | -2.5(-11.7 to 6.7) | -1.4(-12.7 to 9.9) | -6.1(-21.6 to 9.4) | -8.0(-31.3 to 15.3) | -1.3(-11.4 to 8.8) |
| Suriname | 2009-2016 | -8.6(-18.7 to 1.5) | -7.6(-19.7 to 4.5) | -10.2(-27.8 to 7.4) | -25.3(-52.8 to 2.2) | -5.7(-16.5 to 5.1) |
| Trinidad and Tobago | 2011-2017 | -0.7(-8.1 to 6.7) | 1.0(-8.1 to 10.1) | -4.9(-17.5 to 7.7) | 9.5(-6.5 to 25.5) | -2.5(-10.8 to 5.8) |
| Uruguay | 2006-2012 | -0.4(-4.7 to 3.9) | -0.4(-5.5 to 4.7) | 0.7(-6.7 to 8.1) | 1.2(-11.1 to 13.5) | -1.1(-5.7 to 3.5) |
| **Southeast Asia** |  |  |  |  | 18.8(-3.1 to 40.7) | 4.0(-8.5 to 16.5) |
| Indonesia | NA | NA | NA | NA | NA | NA |
| Thailand | 2008-2015 | 6.8(-4.1 to 17.7) | 5.7(-8.2 to 19.6) | 6.6(-10.3 to 23.5) | 18.8(-3.1 to 40.7) | 4.0(-8.5 to 16.5) |
| **Western Pacific** |  |  |  | -4.0(-18.9 to 10.9) | 22.3(-4.5 to 49.1) | 1.1(-8.0 to 10.20) |
| Fiji | 2010-2016 | 8.0(-6.4 to 22.4) | 9.9(-6.4 to 26.2) | 2.5(-27.1 to 32.1) | 0(-10.1 to 10.1) | 7.6(-7.2 to 22.4) |
| Samoa | 2011-2017 | -8.2(-24.8 to 8.4) | -14.7(-36.3 to 6.9) | 3.8(-22.0 to 29.6) | 53.5(38.4 to 68.6)* | -12.9(-30.4 to 4.3) |
| Vanuatu | 2011-2016 | -9.6(-25.2 to 6.0) | -8.3(-27.8 to 11.2) | -17.8(-43.8 to 8.2) | -0.2(-37.7 to 37.3) | -15.0(-31.9 to 1.9) |

**Notes** Data are prevalence (95% Confidence interval). * p<0.05, represent significant downward or upward trends in prevalence.NA: Not Applicable.
